# The Proteolytic Activation, Toxic Effects, and Midgut Histopathology of the *Bacillus thuringiensis* Cry1Ia Protoxin in *Rhynchophorus ferrugineus* (Coleoptera: Curculionidae)

**DOI:** 10.3390/toxins17020084

**Published:** 2025-02-12

**Authors:** Camilo Ayra-Pardo, Victor Ramaré, Ana Couto, Mariana Almeida, Ricardo Martins, José Américo Sousa, Maria João Santos

**Affiliations:** 1 CIIMAR/CIMAR LA, Interdisciplinary Centre of Marine and Environmental Research, University of Porto, Terminal de Cruzeiros do Porto de Leixões, Avda. General Norton de Matos s/n, 4450-208 Matosinhos, Portugal; acouto@fc.up.pt (A.C.); jasousa@fc.up.pt (J.A.S.); 2 Department of Biology, Polytech Clermont, Campus Universitaire des Cézeaux 2 Avenue Blaise Pascal TSA 60206, 63178 Aubière Cedex, France; victor.ramare@sfr.fr; 3 CIIMAR–Biology Department, Faculty of Sciences, University of Porto, 4169-007 Porto, Portugal; mariana.m.almeida07@gmail.com (M.A.); up202009340@edu.fc.up.pt (R.M.)

**Keywords:** biological control, palm pest, insect pathogen, insecticidal crystal proteins, insect bioassays, paraffin histology

## Abstract

The red palm weevil (RPW; Coleoptera: Curculionidae) is a destructive pest affecting palms worldwide, capable of causing significant economic losses and ecological damage in managed palm ecosystems. Current management heavily relies on synthetic insecticides, but their overuse fosters resistance. *Bacillus thuringiensis* (Bt) offers a promising alternative, producing toxins selective against various insect orders, including Coleoptera. However, no specific Bt toxin has yet been identified for RPW. This study investigates the toxicity against RPW larvae of the Bt Cry1Ia protoxin, known for its dual activity against Lepidoptera and Coleoptera. A laboratory RPW colony was reared for two generations, ensuring a reliable insect source for bioassays. Cry1Ia was expressed as a 6xHis-tagged fusion protein in *Escherichia coli* and purified using nickel affinity. Incubation with RPW larval gut proteases for 24 h produced a stable core of ~65 kDa. Diet-incorporation bioassays revealed high Cry1Ia toxicity in neonate larvae. In contrast, the lepidopteran-active Cry1Ac protoxin, used as a robust negative control, was completely degraded after 24 h of in vitro proteolysis and showed no toxicity in bioassays. Cry1Ia-fed larvae exhibited significant midgut cell damage, characteristic of Bt intoxication. These findings highlight Cry1Ia’s strong potential for integration into RPW management programs.

## 1. Introduction

The red palm weevil (RPW) (*Rhynchophorus ferrugineus* Olivier, 1790) (Coleoptera: Curculionidae) is an invasive and highly destructive pest that poses a significant threat to palm species worldwide. Native to Southeast Asia, the RPW has rapidly spread to other regions, including the Middle East, North Africa, and Europe, mainly through the global trade of infested palm plants [1,2]. This pest causes severe damage to a wide range of palm species, including economically important crops such as date palms (*Phoenix dactylifera*), coconut palms (*Cocos nucifera*), and oil palms (*Elaeis guineensis*), as well as ornamental palms like the Canary Island date palm (*Phoenix canariensis*) [3]. The damage is mainly inflicted by the larvae, which burrow into the heart of the palm, feed on its tissues, and result in structural degradation, wilting, and the eventual death of the tree [4]. Beyond its economic impact, RPW disrupts agro-ecosystems where palms play crucial ecological roles, such as providing habitats for wildlife and offering coastal protection [5].

In Mediterranean Europe, RPW was first detected in the early 2000s and has since become a serious problem, particularly in countries with widespread palm cultivation [6]. Spain and Italy have reported substantial infestations, with RPW causing notable economic losses in the ornamental palm sector [7,8]. In Portugal, the spread of RPW is of particular concern, threatening both commercial palm plantations and culturally significant palm species in public and private gardens. The pest has been detected in various regions, with outbreaks concentrated along the southern coast and in Lisbon, where palms are frequently planted in urban landscapes [9,10,11,12,13].

Managing RPW is particularly challenging due to its ability to infest both mature and young palms, coupled with its concealed feeding behavior, which complicates early detection. Chemical insecticides are commonly used to manage RPW [14], yet their effectiveness is often limited, particularly as resistance has been documented in some populations [15]. Additionally, the widespread use of these chemicals raises environmental and human health concerns, reinforcing the need for sustainable and targeted pest management strategies. Other methods, such as pheromone traps for monitoring and physically removing infested palms [16,17], are labor-intensive, costly, and often insufficient to curb RPW spread.

As a result, biological control approaches have gained interest, particularly using entomopathogenic nematodes, fungi, and bacteria [18,19,20,21,22]. Among these, the entomopathogenic bacterium *Bacillus thuringiensis* (Bt) is widely known for its potent insecticidal properties, which stem from the production of crystal proteins (Cry), vegetative insecticidal proteins (Vip), and cytolytic proteins (Cyt) that are specifically active against insect pests from the orders Lepidoptera, Coleoptera, and Diptera, while posing minimal risk to non-target organisms and the environment [23,24]. Cry and Vip proteins have been successfully used in bioinsecticides and transgenic plants engineered for pest resistance [25].

The mechanism by which Cry toxins induce insect mortality is well documented for pest species. Upon ingestion, Cry protoxins are activated by midgut proteases, forming active toxins that pass through the peritrophic matrix and bind to specific receptors on midgut epithelial cells. This binding facilitates oligomerization and membrane insertion, resulting in pore formation that disrupts ion homeostasis, potentially causing osmotic lysis and tissue damage [26]. Alternatively, a programmed cell death (PCD)-centric model proposes that Cry toxins initiate signaling pathways that destabilize ion gradients and disrupt cytoskeletal integrity, ultimately causing cell death [27]. Regardless of the precise mechanism, the result is gut tissue degradation, septicemia, and eventual insect mortality.

To evaluate the efficacy of Bt toxins against RPW, maintaining laboratory colonies is essential for controlled experimental studies. Field-collected populations often vary in age, physiological status, and prior insecticide exposure, which can affect experimental outcomes [22,28,29]. However, maintaining RPW in captivity is challenging due to its long life cycle and specific feeding requirements. Wild-caught insects may also experience physiological stress when exposed to sudden environmental changes—such as confinement, unfamiliar diets, or disrupted ecological cues—which can reduce reproductive efficiency or increase the incidence of unfertilized eggs [30]. Establishing a stable colony is therefore a fundamental step in advancing research on novel control strategies. Traditional rearing methods of RPW rely on sugarcane or palm tissues, which closely mimic the weevil’s natural habitat but pose logistical challenges due to seasonal availability or import costs. To overcome these limitations, artificial diets incorporating essential nutrients can be developed to sustain RPW growth and development while allowing precise experimental control [31,32].

Despite the availability of several Bt Cry toxins active against Coleoptera [33], limited research has been conducted on their efficacy against RPW. Cry3A, the first coleopteran-specific Bt toxin, has shown limited toxicity against *Diabrotica virgifera virgifera* and *Leptinotarsa decemlineata* [34,35]. In RPW larvae, the reported LC_50_ (lethal concentration 50) of Cry3Aa exceeded 4.8 mg/mL, although no exact LC_50_ value or 95% confidence interval was provided [29]. These authors found minimal gut epithelial damage in exposed larvae, with reduced efficacy attributed to Cry3Aa instability in the presence of RPW midgut proteases, likely serine proteases, which degraded the protoxin into potentially inactive fragments [29]. To date, no effective Cry toxin has been identified for RPW.

The Cry1Ia protoxin exhibits dual activity against lepidopteran and coleopteran pests [36], including weevil species within the Curculionidae family as RPW, such as the boll weevil *Anthonomus grandis* [37,38]. On this basis, we hypothesized that Cry1Ia might also be toxic to RPW. To test this, we produced a recombinant Cry1Ia protoxin from Bt var. *kurstaki* (Btk) HD-1 (4D1) in *Escherichia coli* and purified it for bioassays. A laboratory RPW colony was established and maintained on an in-house meridic diet, which must ensure a more reliable insect supply for bioassays compared to using offspring from wild-caught individuals. We assessed Cry1Ia’s stability against RPW gut proteases, its insecticidal activity on neonate larvae, and its effects on RPW midgut epithelial cells. The lepidopteran-specific protoxin Cry1Ac was chosen over Cry3Aa as a robust negative control because it provides a clear, unambiguous baseline for evaluating Cry1Ia’s toxicity to RPW. Our findings confirm Cry1Ia’s insecticidal potential against RPW, supporting its integration into sustainable pest management strategies.

## 2. Results

### 2.1. Establishment of a Laboratory Colony of RPW

**Figure 1:**
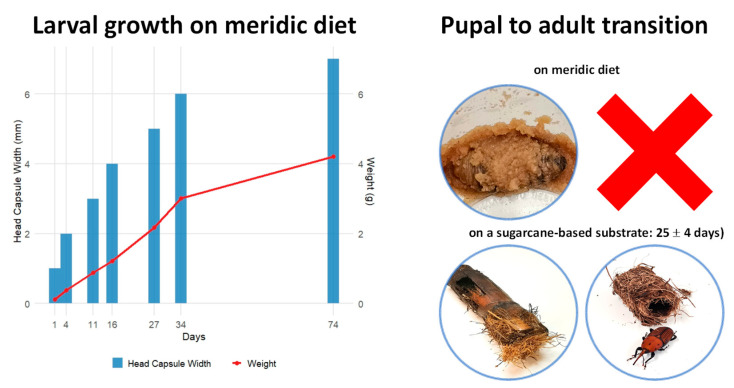
Growth and development of *Rhynchophorus ferrugineus* in a laboratory colony. The graph (**left**) illustrates larval growth on an in-house meridic diet, tracking head capsule width (blue bars) and larval weight (red line) over time. Measurements were recorded after each ecdysis to monitor instar transitions. The images (**right**) depict the pupal-to-adult transition. Larvae reared exclusively on the meridic diet failed to complete development and died (**top-right**, marked with a red cross). In contrast, successful pupation and adult emergence were observed in larvae that were transferred to a sugarcane-based substrate, with weevils completing metamorphosis within 25 ± 4 days (**bottom-right**).

The growth and development of RPW in the laboratory colony were systematically monitored to establish baseline data on larval and pupal stages under controlled rearing conditions. Larval growth on an in-house-developed meridic diet based on wheat germ and corn flour showed a progressive increase in head capsule width and body weight (Figure 1, left). Head capsule width increased by approximately 1 mm after each ecdysis, indicating six molts and seven larval instars over 74 days. The intervals between molts were irregular, with the transition from the 6th to the 7th instar taking significantly longer compared to the previous transitions between instars. Larval weight steadily increased throughout development, with more pronounced gains in the later instars, peaking at approximately 4.5 g during the 7th instar. This sharp weight increase, coupled with the prolonged duration of the final molt, suggests that larvae allocate substantial energy and resources during this phase to prepare for pupation, reflecting the physiological demands of this critical developmental stage. After reaching the 7th instar, larvae began wandering in search of fibers for cocoon construction. Notably, when larvae were left on the diet without being transferred to cages with sugarcane stalks, they failed to construct cocoons and eventually died (Figure 1, top-right). In contrast, when provided with sugarcane, the pupal-to-adult transition occurred successfully, lasting 25 ± 4 days and culminating in adult emergence (Figure 1, bottom-right).

**Figure 2:**
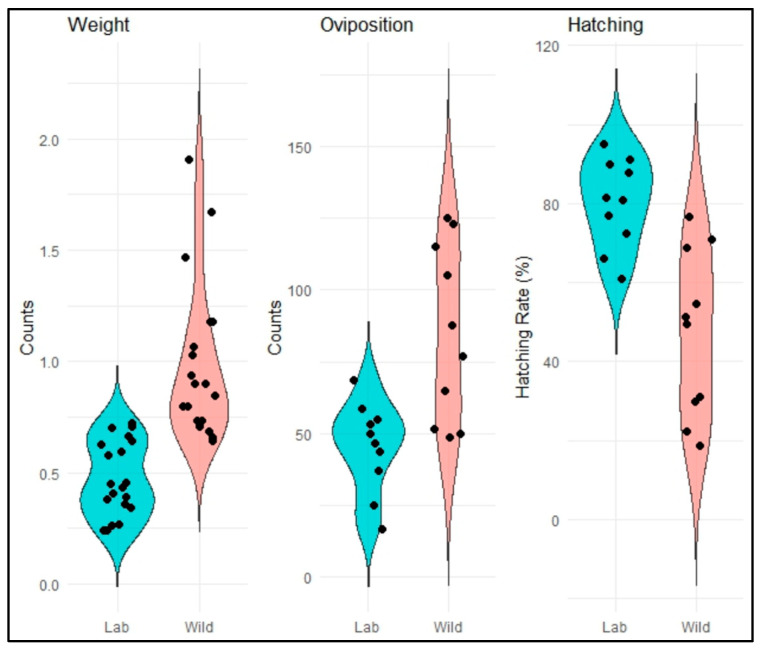
Violin plots comparing adult weight, oviposition, and hatching between wild-captured and laboratory-reared RPW. The data represent adult weights (g), the number of eggs laid, and the percentage of hatched eggs from both sources. Individual data points are shown as black dots within each distribution, and medians are denser regions within the violins. Paired *t*-tests revealed significant differences between populations, with *p* < 0.001 for adult weight, *p* < 0.001 for oviposition, and *p* = 0.002 for hatching rates.

The hypothesis that wild-caught insects are not a reliable source of neonate larvae for bioassays was tested by comparing the weight, fecundity, and fertility of adults from laboratory-reared and wild-caught populations. The results revealed significant differences across all parameters (Figure 2). While wild-caught adults exhibited higher body weights than laboratory-reared individuals (*p* < 0.001) and wild-caught females laid significantly more eggs (*p* < 0.001), the hatching percentage was significantly higher for laboratory-reared insects (*p* = 0.002). The violin plots further illustrate the variability within each group, with narrower distributions in the laboratory-reared population, indicating more uniform conditions in the controlled rearing environment.

### 2.2. Obtainment of Cry1Ia Protoxin

The full-length DNA sequence of the *cry1Ia* gene was amplified by PCR from the Btk strain HD1 (4D1) and cloned into the pHTP1 vector. This vector facilitated the transcriptional fusion of *cry1Ia* with a six-histidine residue tag (6xHis tag) under the control of the strong T7 promoter. The use of the T7 promoter system relies on *E. coli* DE3 strains as host cells, which contain an IPTG-inducible bacteriophage T7 RNA polymerase gene integrated into their genome.

**Figure 3:**
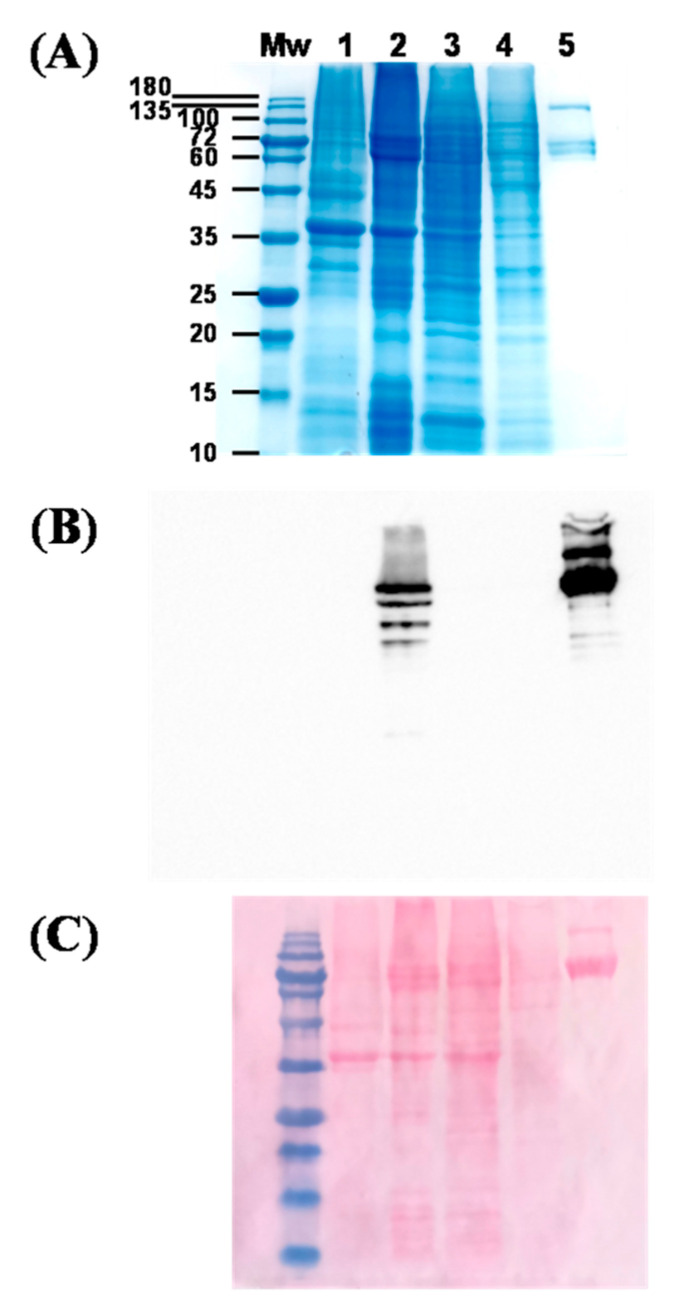
Production and purification of recombinant Cry1Ia protoxin in a His-tag fusion system. (**A**) SDS-10% PAGE analysis of 6xHis-Cry1Ia expression and purification: Lane 1, supernatant of uninduced bacterial cells; Lane 2, supernatant of IPTG-induced bacterial cells after 16 h; Lane 3, flow-through from nickel column (50 mM imidazole); Lane 4, wash fraction (100 mM imidazole); Lane 5, eluted protein (300 mM imidazole). Mw, molecular weight marker: NZYBlue protein marker (10–180 kDa) (NZYTech, Lisbon, Portugal). (**B**) Western blot of electrophoresed proteins blotted onto 0.45 µm nitrocellulose membranes and probed with a rabbit anti-6xHis polyclonal antibody conjugated to HRP (Abcam, Cambridge, UK). The conjugate was diluted 1:5000 in TBST (50 mM Tris, pH 7.6, 150 mM NaCl, 0.2% Tween 20), and the blot was developed with Pierce ECL Western blotting Substrate (Thermo Fisher Scientific, Hanover Park, IL, USA). (**C**) Ponceau S (0.1%) reversible staining of the blot.

Recombinant expression and nickel affinity purification of 6xHis-Cry1Ia were pursued by SDS-10% PAGE and Western blotting (Figure 3). To optimize soluble protein production, bacterial cultures were grown at 18 °C, as Cry1Ia predominantly accumulated in the insoluble fraction at 37 °C. A product of ~82 kDa, consistent with the predicted size of 6xHis-Cry1Ia, was detected in the soluble protein fraction of IPTG-induced bacterial cells grown for 16 h and was absent in uninduced cultures (Figure 3A, lanes 1 and 2). This product was unequivocally recognized by anti-His-tag polyclonal HRP-conjugated antibodies (Figure 3B,C, lane 2).

Further purification steps confirmed the high specificity of the binding to the nickel resin. The absence of the ~82 kDa band in the flow-through (Figure 3A–C, lane 3) and the wash fraction (100 mM imidazole; Figure 3A–C, lane 4) indicates strong binding to the nickel resin. Proteins lacking the 6xHis tag and weakly bound proteins were removed during washing. Elution with 300 mM imidazole yielded a highly pure ~82 kDa product, as confirmed by anti-His-tag HRP detection (Figure 3A–C, lane 5).

### 2.3. Incubation of Cry1Ia Protoxin with RPW Midgut Proteases

**Figure 4:**
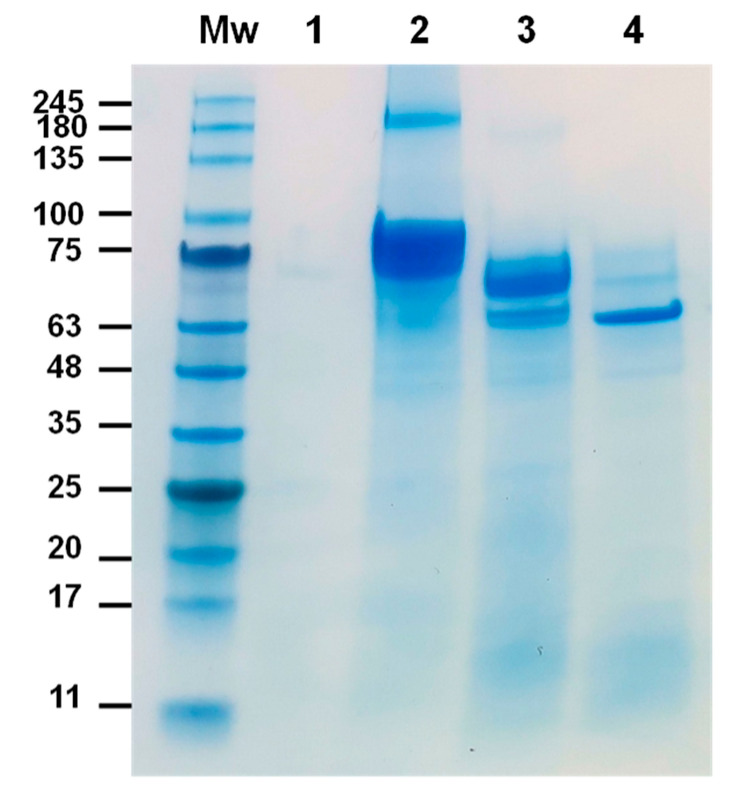
SDS-PAGE analysis of proteolytic activation of Cry1Ia (A) protoxin by RPW (third instar) gut proteases. Lane 1: gut juice in carbonate buffer (5% [*v*/*v*]) after 24 h incubation at 37 °C; Lane 2: purified Cry1Ia protoxin (1 μg); Lane 3: Cry1Ia processed product after 1 h incubation at 37 °C with gut juice (5% [*v*/*v*]); Lane 4: Cry1Ia processed product after 24 h incubation at 37 °C with gut juice (5% [*v*/*v*]). Mw, molecular weight marker (kDa): NZYColour Protein Marker II (11–245 kDa) (NZYTech, Lisbon, Portugal).

The proteolytic processing of Cry1Ia protoxin by RPW gut proteases was analyzed using in vitro digestion assays. As illustrated in Figure 4, the ~82 kDa Cry1Ia protoxin underwent controlled enzymatic cleavage upon exposure to gut juice (5% [*v*/*v*]). After 1 h, the protoxin was primarily converted into an intermediate product of ~70 kDa, with a minor fraction further processed into a stable ~65 kDa core fragment. By 24 h, the ~65 kDa product was the sole detectable band, demonstrating its resistance to further proteolysis. In contrast, a recombinant lepidopteran-active Cry1Ac protoxin used as robust negative control—obtained from the Bacillus Genetic Stock Center (BGSC, Cat. No. ECE53) and purified by a standard procedure as described below in Section 4.5—exhibited rapid and extensive degradation by RPW gut proteases, with no detectable products observed on the SDS-PAGE gel after 24 h.

LC-MS/MS analysis verified the identity of the ~65 kDa fragment as a processed variant of Cry1Ia protein (UniProtKB/Swiss-Prot: Q45752). Among the identified peptides, [K].VTALFTSTNPR.[G] was mapped to positions 660–670 in the Cry1Ia sequence. No tryptic peptides were detected beyond position 670, strongly suggesting that this represents the C-terminal end of Cry1Ia after digestion by RPW gut proteases. This peptide was identified with high confidence (Percolator q-value: 0.0006433; Percolator PEP: 0.002088; XCorr: 3.3) and in high abundance (LFQ-scaled intensity: 100), with a theoretical monoisotopic mass of 1206.64771 Da, confirming it as the C-terminal boundary of the processed protein.

Another peptide, [K].GKNQWEIFMEHVEEIINQK.[I], was detected with high confidence (Percolator q-value: 0.0006433; Percolator PEP: 0.0008692; XCorr: 2.9) and mapped to positions 97–115 in the Cry1Ia sequence. It was also identified in high abundance (LFQ-scaled intensity: 100) with a theoretical monoisotopic mass of 2372.17034 Da. Notably, this peptide contained one missed tryptic cleavage site (K96–K98), yet no peptides were detected upstream. The absence of upstream peptides strongly suggests that RPW gut proteases cleaved Cry1Ia up to K96, defining this region as the N-terminal boundary of the processed protein. Based on these sequence boundaries, the inferred molecular weight of the processed Cry1Ia is 64.5 kDa, aligning with the size reduction observed in SDS-PAGE (Figure 4).

### 2.4. Toxicity of Cry1Ia Protoxin to RPW Larvae

**Figure 5:**
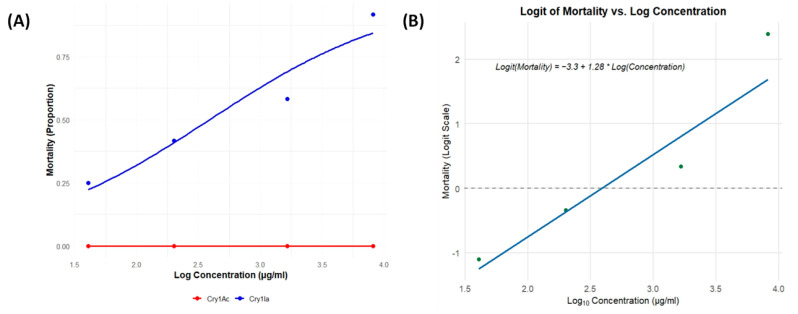
Mortality of RPW neonate larvae in bioassays with Bt Cry1Ia and Cry1Ac protoxins. (**A**) The mortality (proportion) is plotted against the log-transformed concentration (µg/mL) for each protoxin. Data points represent observations from 10-day mortality assays. The blue line depicts the predicted values from the minimal adequate statistical model fitted to the Cry1Ia bioassay data, demonstrating a significant dose-dependent response. In contrast, the red line shows no insecticidal activity for Cry1Ac, with mortality consistently near zero across all tested concentrations. (**B**) Logit regression analysis of Cry1Ia protoxin data. The relationship between log-transformed concentration and mortality (on a logit scale) is described by the equation Logit (Mortality) = −3.3 + 1.28 × Log (Concentration). The dashed line represents the LC_50_ value, indicating the concentration required to achieve 50% mortality.

Bioassays assessing the insecticidal activity of Bt Cry1Ia protoxin against neonate RPW larvae revealed a significant dose-dependent mortality response, whereas Cry1Ac, used as a robust negative control, caused no mortality, comparable to the non-exposed controls. Figure 5 shows (A) the relationship between larval mortality (proportion) and log-transformed protoxin concentrations and (B) the logit regression analysis of the Cry1Ia bioassays.

A logistic regression model was applied to the Cry1Ia bioassay data, identifying log (concentration) as a significant predictor of mortality with an estimated slope (coefficient) of 1.276 (S.E. = 0.410, z = 3.109, *p* = 0.002). The intercept was estimated at −3.303 (S.E. = 1.149, z = −2.875, *p* = 0.004), representing the log odds of mortality when the concentration is at the reference level. The model demonstrated a good fit, with a null deviance of 13.227 (3 degrees of freedom) and a residual deviance of 1.231 (2 degrees of freedom). The Akaike Information Criterion (AIC) value was 15.757. The Fisher scoring algorithm converged in four iterations.

Based on the logistic regression model, we estimated the LC_50_, LC_90_, and LC_10_ values of Cry1Ia for neonate RPW larvae and their 95% confidence intervals. These metrics are critical for assessing Cry1Ia’s toxicity, efficacy, and potential for commercial application as an insecticidal agent against RPW. On the log-transformed scale, the estimated LC_50_ was 2.59, corresponding to 13.32 µg/mL on the original scale. The 95% confidence interval for LC_50_ ranged from 7.98 to 22.23 µg/mL, indicating the concentration required to achieve 50% mortality with 95% confidence. On the original scale, LC_90_ and LC_10_ were estimated at 74.58 µg/mL (95% CI: 44.70–124.45) and 2.38 µg/mL (95% CI: 1.43–3.97), respectively.

### 2.5. Intoxication with Cry1Ia Protoxin Damaged the Gut Epithelium of RPW Larvae

**Figure 6:**
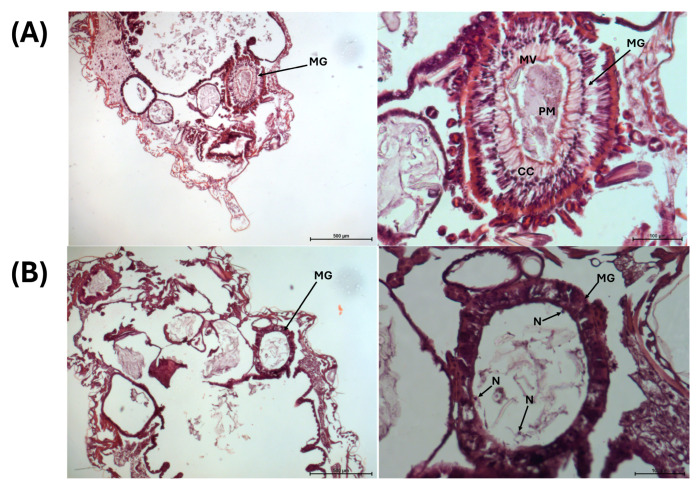
Histological analysis of RPW neonate larvae following 48 h of Cry1Ia treatment. Paraffin histology sections of larvae, which were cut longitudinally, were stained to assess gut morphology. (**A**) Untreated larvae: Left: Broad view showing organized abdominal tissues and intact midgut structure. Right: Close-up of the midgut epithelium, displaying normal cellular architecture, including a well-defined brush border and intact peritrophic membrane. (**B**) Cry1Ia-treated larvae: Left: Broad view revealing disrupted abdominal tissues with extensive structural damage. Right: Close-up of the midgut epithelium, showing severe epithelial degradation characterized by disorganized cellular layers, loss of the brush border and peritrophic membrane, and nuclei from lysed cells visible in the gut lumen. CC: columnar cells; MG: midgut; MV: microvilli; N: nuclei; PM: peritrophic membrane enveloping the gut content. Scale bars: 500 µm (broad views) and 100 µm (close-ups).

Histological examination of larvae exposed to 13 μg/mL of Cry1Ia protoxin (a concentration equivalent to the LC_50_) for 48 h revealed significant morphological differences compared to untreated (control) RPW larvae. In untreated larvae (Figure 6A), the gut epithelium exhibited a well-preserved brush border, with columnar cells showing normal organization and no signs of structural compromise. In contrast, Cry1Ia-treated larvae (Figure 6B) displayed severe disruption of the gut epithelium, characterized by the loss of cellular integrity, disorganization of the midgut structure and peritrophic membrane, and nuclei from lysed cells visible in the gut lumen. These findings suggest that Cry1Ia toxin induces extensive gut damage, consistent with its proposed mode of action targeting midgut epithelial cells [38].

## 3. Discussion

In this study, we demonstrated that Cry1Ia protein from Btk HD1 is toxic to RPW and holds potential for RPW control strategies. Establishing a laboratory RPW colony on an in-house meridic diet was essential to ensure a reliable source of high-quality insects for bioassays. While natural diets such as sugarcane and date palm tissues closely mimic RPW’s natural environment, their limited local availability and the necessity of importation, in our case, made them economically impractical. Additionally, the meridic diet enabled precise blending of toxins at different concentrations, ensuring consistency in bioassays.

RPW development time and larval instar progression are closely linked to the feeding substrate [32]. We observed a steady increase in larval head capsule width (~1 mm per molt) and used this parameter to estimate instar numbers. After the 7th instar, larvae began wandering in search of fibers for cocoon formation and were transferred to a box with 10–15 cm long sugarcane stalk. Larvae not transferred in time died on the diet, reinforcing the necessity of fibers for metamorphosis in RPW. However, some larvae failed to form cocoons even in the presence of sugarcane, resulting in adults with shortened elytra and exposed abdominal segments, leading to early mortality (Figure S1). This suggests that a cocoon is essential for the full extension and hardening of elytra during metamorphosis in RPW.

Significant reproductive differences emerged between laboratory-reared and wild-caught RPW populations. Wild adults laid more eggs, yet a high percentage failed to hatch, whereas laboratory-reared RPW exhibited significantly higher hatching rates, suggesting some adaptation to confined rearing conditions [39]. To better understand these adaptive processes, we aim to collect data across generations to develop a mathematical model describing RPW population growth and development on our meridic diet under controlled conditions. Since a reliable model requires sufficient generational data, ongoing monitoring is crucial to capturing population dynamics, generational turnover, and potential adaptations, ultimately improving the predictive power of laboratory studies for field applications.

Our bioassay data revealed that Cry1Ia protoxin induced high mortality in neonate RPW larvae, with an LC_50_ of 13.32 μg/mL (95% CI: 7.98–22.23), confirming strong insecticidal activity against this pest. Surviving larvae exhibited stunted growth and delayed molting, in stark contrast to the normal development observed in larvae exposed to the non-toxic Cry1Ac protein or unexposed controls. These findings align with reports of Cry1Ia toxicity against another Curculionidae species, *A. grandis* (cotton boll weevil), which had an LC_50_ of 21.5 μg/mL (95% CI: 17.0–26.0) [38]. Notably, these authors described Cry1Ia as approximately 15 times more toxic to *A. grandis* than Bt var. *tenebrionis*, which expresses Cry3Aa. A recent study found that the Bt insecticidal proteins Mpp23Aa/Xpp37Aa (formerly Cry23Aa/Cry37Aa) were highly toxic to *A. grandis* larvae, with an estimated LC_50_ of 0.18 μg/g diet [40]. These proteins also exhibited toxicity against other coleopteran pests, including *Tribolium castaneum* [41], *Cylas* spp. [42], and *Xylotrechus Arvicola* [43]. Investigating the toxicity of Mpp23Aa/Xpp37Aa against RPW could be an interesting avenue for future research.

The LC_90_ and LC_10_ values reported in this study offer essential insights into the efficacy of Cry1Ia against RPW. The LC_90_ of 74.58 µg/mL (95% CI: 44.70–124.45), representing the concentration required to achieve 90% mortality, underscores the Cry1Ia concentration for effective pest control. While this value was derived under controlled laboratory conditions, formulation optimization and the inclusion of synergistic compounds could further enhance Cry1Ia’s effectiveness in field applications. The LC_10_ value of 2.38 µg/mL (95% CI: 1.43–3.97), representing the concentration at which 10% mortality is observed, is particularly significant for evaluating sublethal effects on behavior, growth, and reproduction. Such effects could influence RPW population dynamics, feeding patterns, and interactions with natural enemies, thereby contributing to a more comprehensive ecological understanding of Cry1Ia’s broader impact beyond direct mortality.

In our study, histopathological analysis provided critical insights into the mode of action of Cry1Ia in RPW larvae, demonstrating significant midgut epithelial damage following exposure. Although the Cry1Ia concentration used for histopathology corresponded to the LC_50_ from bioassays, all analyzed samples consistently exhibited complete gut destruction, highlighting the potent activity of Cry1Ia in inducing cell lysis. Key observations included the disintegration of brush border integrity and the extrusion of cellular contents, including nuclei, into the gut lumen—hallmarks of Cry toxin activity. The severe cellular disruption compromises the midgut barrier, impairing digestion, nutrient absorption, and overall gut function. Additionally, the loss of the peritrophic membrane, a protective barrier encasing gut contents [44,45], exacerbates the damage by exposing the epithelial layer to gut enzymes, pathogens, and environmental stressors. Notably, the destruction of the gut epithelium permits gut bacteria to invade the hemolymph, proliferate, and induce insect death via septicemia. These effects are typically initiated by the binding of the toxin to specific receptors on midgut epithelial cells [23]. While the receptor for Cry1Ia in RPW or other coleopteran species remains unidentified, studies on *Spodoptera* spp. (Lepidoptera) have described a potential receptor as a 65 kDa polypeptide [46].

Proteolytic activation by insect gut proteases plays a critical role in the mode of action of Bt Cry toxins, facilitating the conversion of the protoxin into its active form [47,48]. This process is pivotal for the toxin’s potency and can also influence its specificity toward target insect species and the development of resistance mechanisms [49,50]. In our study, the 81 kDa Cry1Ia protoxin was processed to a stable core fragment of ~65 kDa after 24 h of incubation with insect gut juice. This stability suggests that Cry1Ia undergoes controlled and selective proteolysis, leading to the generation of a robust and functional toxin core capable of exerting its insecticidal effects on the midgut epithelium. Interestingly, a similar ~65 kDa polypeptide was previously detected in *Spodoptera* sp. infected with a recombinant baculovirus engineered to express the Cry1Ia protoxin [38]. This polypeptide was absent when insects were infected with either wild-type baculovirus or in mock-infected control and was specifically recognized by anti-Cry1Ia antibodies, confirming it as the processed, active form of Cry1Ia. In contrast, the Cry3Aa protoxin demonstrated a different proteolytic profile when exposed to RPW gut proteases. Instead of yielding the 55 kDa active fragment expected for this kind of toxin, Cry3Aa was excessively hydrolyzed, resulting in smaller fragments (<45 kDa) [29]. This over-degradation likely compromises its insecticidal efficacy, potentially explaining the absence of toxicity.

Our results suggest that Cry1Ia could be integrated into RPW control programs through different biotechnological approaches. One potential strategy involves engineering endophytic bacteria to express Cry1Ia, allowing colonization of palm tissues and providing sustained protection against RPW infestation. Another avenue for exploration is the development of transgenic palms expressing Cry1Ia to extend pest control throughout the season. Both approaches take advantage of the selective toxicity and environmental safety of Cry proteins. Given that neonate larvae are the most susceptible stage, targeting them would likely maximize efficacy. However, further research is needed to assess Cry1Ia toxicity in older instars to fully evaluate its potential against RPW.

In summary, our findings demonstrate that Cry1Ia is highly toxic to RPW, with its insecticidal activity reinforced by its remarkable stability against degradation by RPW gut proteases. The extensive midgut epithelial damage observed further highlights the intensity of Cry1Ia’s action, confirming its potent mode of action against this pest. These results support the integration of Cry1Ia into RPW management programs, particularly in combination with complementary technologies, as a promising alternative to conventional control strategies.

## 4. Materials and Methods

### 4.1. Insects

A laboratory colony of RPW was established at the Faculty of Sciences, University of Porto, using eggs laid by adult weevils captured in pheromone traps in the Porto region (41.147716, −8.670845), Portugal. Adult weevils were paired by sex, provided with apple slices, and housed in plastic cages to facilitate mating and oviposition. Collected eggs were placed on cotton wool soaked in a 20% honey solution within labeled containers and incubated in a controlled environment chamber at 28 ± 1 °C, 80 ± 5% relative humidity, and a 16:8 (light:dark) photoperiod.

Hatched larvae were reared on a wheat germ/corn flour-based meridic diet (Table 1) supplemented with an apple smoothie as a token stimulus. Larvae were monitored during development by recording weight and head capsule width at each molting stage. Upon reaching the seventh instar, sugarcane stalks were provided to facilitate cocoon formation and pupation.

Twenty emerging weevils (ten females and ten males) were weighed, paired by sex, and housed separately in cages provided with apple slices to facilitate mating and oviposition. The cages were inspected daily for eggs, which were collected, counted, and incubated under the previously described conditions until hatching. Neonate larvae were returned to the diet to sustain the rearing cycle. The colony was maintained under these standardized conditions for at least two generations prior to conducting bioassays, ensuring consistency and a reliable supply of larvae.

### 4.2. Cloning of Cry1Ia Gene

The Btk HD1 strain carrying the *cry1Ia* gene was obtained from the BGSC (Cat. no. 4D1). Bacterial DNA was extracted using the NZY Tissue gDNA Isolation Kit (NZYTech, Lisbon, Portugal) with a lysozyme pretreatment step, following the manufacturer’s instructions. The *cry1Ia* gene was amplified via PCR using mutagenic primers CRY1I-F (5′-TCAGCAAGGGCTGAGGATGAAACTAAAGAATCAAG-3′) and CRY1I-R (5′-TCAGCGGAAGCTGAGGCATGTTACGCTCAATATGG-3′), which included 16 bp overhangs at their 5′ ends (underlined) to provide vector-compatible single-strand termini. These overhangs enabled seamless integration into the pHTP1 vector included in the NZYEasy Cloning & Expression Kit I (NZYTech, Lisbon, Portugal).

PCR amplification and cloning were confirmed by electrophoresis on 1.0% agarose gels stained with GreenSafe Premium (NZYTech, Lisbon, Portugal). The gels were visualized under UV light using a Gel Doc™ XR+ imaging system (Bio-Rad, Hercules, CA, USA). Recombinant pHTP1-Cry1Ia clones were confirmed by sequencing performed at STAB Vida (Lisbon, Portugal).

### 4.3. Expression and Purification of 6xHis-Cry1Ia Protoxin

The recombinant plasmid pHTP1-Cry1Ia was transformed into the *E. coli* BL21 (DE3) strain (NZYTech, Lisbon, Portugal) using the standard heat-shock method. Transformed bacteria were cultured in Luria–Bertani (LB) medium supplemented with 50 µg/mL kanamycin at 37 °C with shaking. When the optical density at 600 nm (OD_600_) reached 0.4–0.6 (mid-log phase), expression of the 6xHis-Cry1Ia protoxin was induced by adding IPTG to a final concentration of 0.2 mM. Induced cultures were incubated for 16 h at 18 °C with shaking.

After induction, 50 mL of the culture was harvested by centrifugation at 3500× *g* for 10 min at 4 °C. The resulting cell pellet was resuspended in 5 mL of NZY Bacterial Cell Lysis Buffer (NZYTech, Lisbon, Portugal) and swim rocket for 30 min to facilitate gentle cell wall disruption. The lysate was clarified by centrifugation at 14,000× *g* for 20 min at 4 °C, and the supernatant was filtered through a 0.22 µm filter. Imidazole was added to the clarified lysate to a final concentration of 50 mM before loading it onto a HisPur Ni-NTA Spin Column (Thermo Fisher Scientific, Hanover Park, IL, USA) pre-equilibrated with lysis buffer containing 50 mM imidazole for immobilized metal affinity chromatography (IMAC) purification.

The column was washed three times with six column volumes of washing buffer (1× PBS, 100 mM imidazole) to remove non-specifically bound proteins. Elution was carried out using 1× PBS containing 300 mM imidazole. Aliquots (12 µL) of the eluted fractions were mixed with 3 µL of 5× SDS-PAGE Sample Loading Buffer (NZYTech, Lisbon, Portugal), boiled for 3 min, and analyzed by reducing sodium dodecyl sulfate-polyacrylamide gel electrophoresis (SDS-PAGE, 10%) following the protocol of Laemmli [51]. Gels were stained with BlueSafe (NZYTech, Lisbon, Portugal) for visualization or used for Western blotting in Section 4.4.

Residual imidazole was removed from the purified 6xHis-Cry1Ia protein, and the buffer was changed to carbonate using PD-10 desalting columns (Cytiva, MA, USA). Protein concentration was determined using the Pierce BCA Protein Assay Kit (Thermo Fisher Scientific, Hanover Park, IL, USA) with bovine serum albumin (BSA) as the standard.

### 4.4. Western Blotting

Western blotting was performed to confirm the purification of the recombinant Cry1Ia protoxin. Proteins separated on a 10% SDS-PAGE gel were transferred onto a nitrocellulose membrane (GE Healthcare, Chicago, IL, USA) using a wet transfer system operated at 100 V for 1 h at 4 °C. Transfer efficiency was assessed by briefly staining the membrane with 0.1% Ponceau S, followed by washing with distilled water to remove the stain prior to immunodetection. The membrane was blocked with 5% (*w*/*v*) non-fat milk powder in Tris-buffered saline containing Tween 20 (TBST: 50 mM Tris, pH 7.6, 150 mM NaCl, 0.2% Tween 20) for 30 min at room temperature with constant shaking. It was then incubated with a rabbit polyclonal anti-6xHis tag antibody conjugated to horseradish peroxidase (1:2000 dilution; Abcam, Cambridge, UK) prepared in TBST. Following primary antibody incubation, the membrane was washed three times with TBST to remove unbound antibodies. Signal detection was carried out using the Pierce ECL Western blotting substrate (Thermo Fisher Scientific, Hanover Park, IL, USA) according to the manufacturer’s protocol.

### 4.5. Obtainment of Cry1Ac Protoxin

In our laboratory, we routinely produce Cry1A-subclass protoxins using recombinant clones obtained from the BGSC. The protoxins are purified through selective solubilization of inclusion bodies containing Cry proteins, using a carbonate buffer (50 mM Na_2_CO_3_, 100 mM NaCl, pH 9.6) supplemented with 10 mM dithiothreitol (DTT), following the standard procedure previously described in [52]. For Cry1Ac, a transformant of *E. coli* strain JM103 carrying the *cry1Ac* gene cloned into the expression vector pKK223-3 was obtained from the BGSC (Cat. no. ECE53). The recombinant strain was cultured in 50 mL of LB medium supplemented with 100 µg/mL ampicillin and induced with 1 mM IPTG. A culture growing in the absence of the inducer was used as the negative control. Cultures were incubated for 16 h at 37 °C with shaking. Following incubation, cells were harvested by centrifugation at 3500× *g* for 10 min at 4 °C. The pellet was resuspended in 5 mL of NZY Bacterial Cell Lysis Buffer (NZYTech, Lisbon, Portugal) to lyse the cells. The insoluble fraction, containing Cry1Ac inclusions, was isolated by centrifugation at 13,000× *g* for 20 min at 4 °C, and the supernatant was discarded. Cry1Ac inclusions were solubilized in carbonate buffer supplemented with DTT at 37 °C for 60 min. The samples were subsequently centrifuged at 13,000× *g* for 20 min, and the soluble fractions were isolated. The solubilized Cry1Ac protoxin was visualized in SDS-PAGE (10% gel) stained with BlueSafe (NZYTech, Lisbon, Portugal) (Figure S2), and the concentration was determined by densitometry using a ChemiDoc imaging system (Bio-Rad, Hercules, CA, USA), with BSA standards for calibration.

### 4.6. Midgut Juice Isolation and in Vitro Processing of Protoxins

Fifth-instar RPW larvae were chilled on ice for 10 min to facilitate tissue dissection. Midgut tissues were carefully isolated, and midgut juice was extracted by centrifugation at 14,000× *g* for 20 min at 4 °C. The resulting supernatant was transferred to a fresh tube and subjected to a second centrifugation under identical conditions to enhance clarity. The final supernatant was filtered through 0.22-μm filters to eliminate residual particulates and stored at −80 °C until further use.

For in vitro protoxin processing, 5 μg of purified 6xHis-Cry1Ia or solubilized Cry1Ac protoxins were incubated with gut juice (5% [*v*/*v*] at 37 °C for 24 h. Proteolysis was terminated by adding phenylmethylsulfonyl fluoride (PMSF) to a final concentration of 1 mM. To assess protoxin digestion, 10 μL samples were analyzed by SDS-PAGE (10% gel), followed by staining with BlueSafe (NZYTech, Lisbon, Portugal) for visualization. Proteolyzed product bands were excised from the gels and analyzed via liquid chromatography-tandem mass spectrometry (LC–MS/MS) at the Proteomics Platform of the i3S—Instituto de Investigação e Inovação em Saúde, Porto, Portugal (https://www.i3s.up.pt/scientific-platform.php?groupid=56, accessed on 1 December 2024). The detected peptide sequences were aligned to identify the proteolysis product and estimate its boundaries and putative C-terminal cleavage site. Peptide quantification was carried out by label-free quantification.

### 4.7. Insect Bioassays

Insect bioassays were conducted using the diet incorporation method to evaluate the insecticidal effects of the 6xHis-Cry1Ia protoxin against RPW larvae. An artificial diet was prepared, and the protoxin was incorporated at four concentrations: 100, 50, 25, and 5 μg/mL. Each concentration was tested against neonate larvae (≤24 h old), using three replicates per treatment with 12 larvae per replicate. Larvae were reared on a treated diet under controlled conditions (temperature: 28 ± 1 °C, relative humidity: 80 ± 5%, photoperiod: 16:8 [L:D]) for 10 days. At the end of the bioassay, mortality was assessed by recording the number of dead and alive larvae in each treatment. The larvae were considered dead if they showed no movement upon gentle probing. The Cry1Ac protoxin (ECE53) obtained in Section 4.5 was used as a robust negative control. Mortality data were used for the bioassay analysis, as described in Section 4.9.

### 4.8. Histopathological Studies

Neonate RPW larvae were exposed to a Cry1Ia protoxin concentration of 13 µg/mL, corresponding to the LC_50_ value, for two days before being used for histopathological analysis. Larvae were rinsed in phosphate-buffered saline (1× PBS, pH 7.4) and fixed in 10% neutral buffered formalin at room temperature for 48 h. The fixed larvae were processed using a tissue processor Citadel 2000 (Thermo Fisher Scientific, Hanover Park, IL, USA), including dehydration through a graded ethanol series (70%, 80%, 90%, and 100%), clearing in xylene, and embedding in paraffin wax. The paraffin blocks were cut into 4–5 μm thick slices comprising longitudinal cuts of the larvae using a rotary microtome and mounted on glass slides. The sections were deparaffinized in xylene, rehydrated through a descending ethanol series, and stained with hematoxylin and eosin (H&E) in a slide stainer Shandon Varistain 24-4 (Thermo Fisher Scientific, Hanover Park, IL, USA) for general histological examination. Slides were mounted with DPX mounting medium.

The stained sections were examined under a light microscope at various magnifications to assess the structural integrity of the midgut epithelium, focusing on the columnar cells, the primary targets of Bt Cry toxins. Representative images were captured using an Axiophot microscope (Carl Zeiss, Oberkochen, Germany) connected to a computer running Leica Application Suite X (LAS X) image analysis software (version 5.1.0). Untreated larvae of the same age served as a control group and provided the basic histological data of the healthy midgut tissue. Comparative analyses between treated and control groups were performed to evaluate the specific effects of Cry1Ia protoxin exposure.

### 4.9. Statistics

To compare the weights, oviposition counts, and hatching rates between the adults captured in the wild “Wild” and from the laboratory colony “Lab”, a paired *t*-test was performed. Statistical significance was evaluated at *p* < 0.05.

For bioassay analysis, a logistic regression model was applied to the mortality data using R software (R Core Team, version 4.3.3) [53]. Mortality proportions were calculated as the ratio of dead larvae to the total number of larvae per concentration. Logistic regression was performed with the generalized linear model (GLM) function (glm) with a binomial family and a log-transformed concentration as the predictor variable. The quasibinomial was used to adjust for overdispersion. The LC_50_, LC_90,_ and LC_10_ values were derived using the dose.p function in R package MASS [54], and their variance and standard error were calculated from the model coefficients and variance–covariance matrix. Overall, 95%CI for each LC_50_, LC_90,_ and LC_10_ were determined using the standard error and the quantile of the normal distribution. Back-transformation to the original concentration scale was performed to report LC_50_, LC_90,_ and LC_10_ values and their 95%CI in the original units. The statistical model was visualized using the base R plotting function plot [53].

## Figures and Tables

**Table 1:** Composition and quantities of ingredients for 1.0 L of RPW diet.

Ingredient	Quantity
Distilled water	900 mL
Agar	15.0 g
Wheat germ	45.0 g
Maize flour	45.0 g
Brewer’s yeast	7.0 g
Vitamin mix	6.0 g
Ascorbic acid	4.0 g
Methylparaben	3.3 g
Fabco-I	2.5 g
Sorbic acid	1.6 g
Apple smoothie	100 g

## Data Availability

The original contributions presented in this study are included in this article and Supplementary Material. Further inquiries can be directed to the corresponding authors.

## Supplementary Materials

The following supporting information can be downloaded at: https://www.mdpi.com/article/10.3390/toxins17020084/s1, Figure S1: Unusual pupal-adult transition and adult phenotype in *Rhynchophorus ferrugineus* (Olivier, 1790); Figure S2: SDS-10% PAGE analysis of Cry1Ac protoxin expression and solubilization.
