# Supplementary material for: The Proteolytic Activation, Toxic Effects, and Midgut Histopathology of the Bacillus thuringiensis Cry1Ia Protoxin in Rhynchophorus ferrugineus (Coleoptera: Curculionidae)
Source: Toxins (Basel). 2025 Feb 12;17(2):84. doi: 10.3390/toxins17020084 (PMC11861718; doi:10.3390/toxins17020084)

Supplementary Figure S1. Unusual pupal-adult transition and adult phenotype in *Rhynchophorus ferrugineus* (Olivier, 1790). Representative images depict the atypical transition outside the cocoon (left) and an emerged adult exhibiting the rare short elytra phenotype (right).

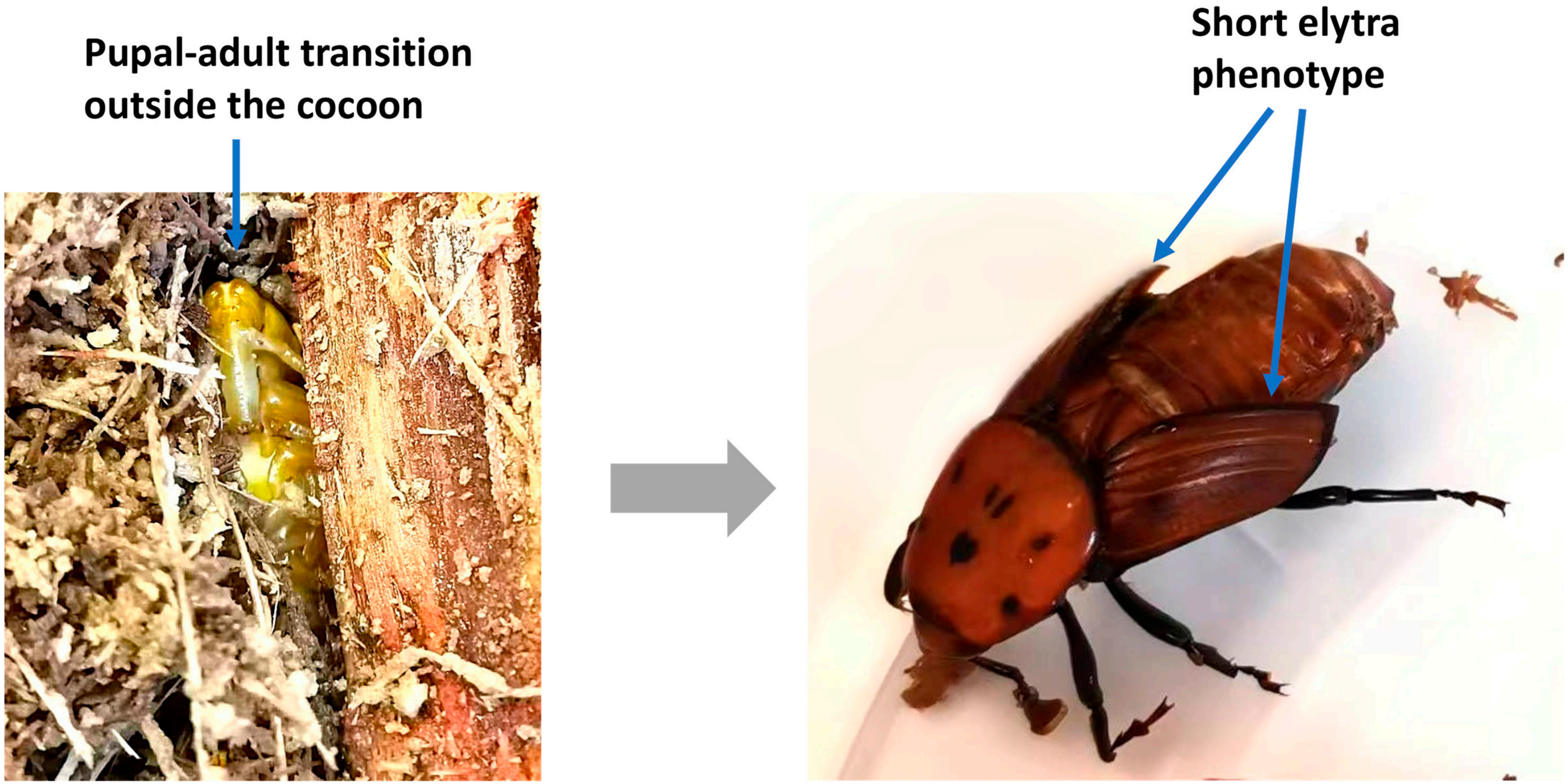

Supplement: Supplementary file 1 [file toxins-17-00084-s001.zip › Supplementary Figure S1.pdf]
