# Supplementary material for: The Proteolytic Activation, Toxic Effects, and Midgut Histopathology of the Bacillus thuringiensis Cry1Ia Protoxin in Rhynchophorus ferrugineus (Coleoptera: Curculionidae)
Source: Toxins (Basel). 2025 Feb 12;17(2):84. doi: 10.3390/toxins17020084 (PMC11861718; doi:10.3390/toxins17020084)

Supplementary Figure S2. SDS-10% PAGE analysis of Cry1Ac protoxin expression and solubilization. (A) Total cell proteins of BGSC ECE53 bacterial clone before (-IPTG) and after (+IPTG) induction with IPTG (Isopropyl β-D-1-thiogalactopyranoside). A distinctive band of ~135 kDa, corresponding to the full-length Cry1Ac protoxin, is visible only in the +IPTG fraction. Molecular weight markers (MM) are shown on the left (kDa). (B) Solubilization of Cry1Ac inclusions in carbonate buffer (50 mM Na_2CO_3, 100 mM NaCl, pH 9.6) supplemented with 10 mM dithiothreitol (DTT) under standard *in vitro* conditions. The ~135 kDa Cry1Ac protoxin band in the +IPTG treatment confirms efficient solubilization.

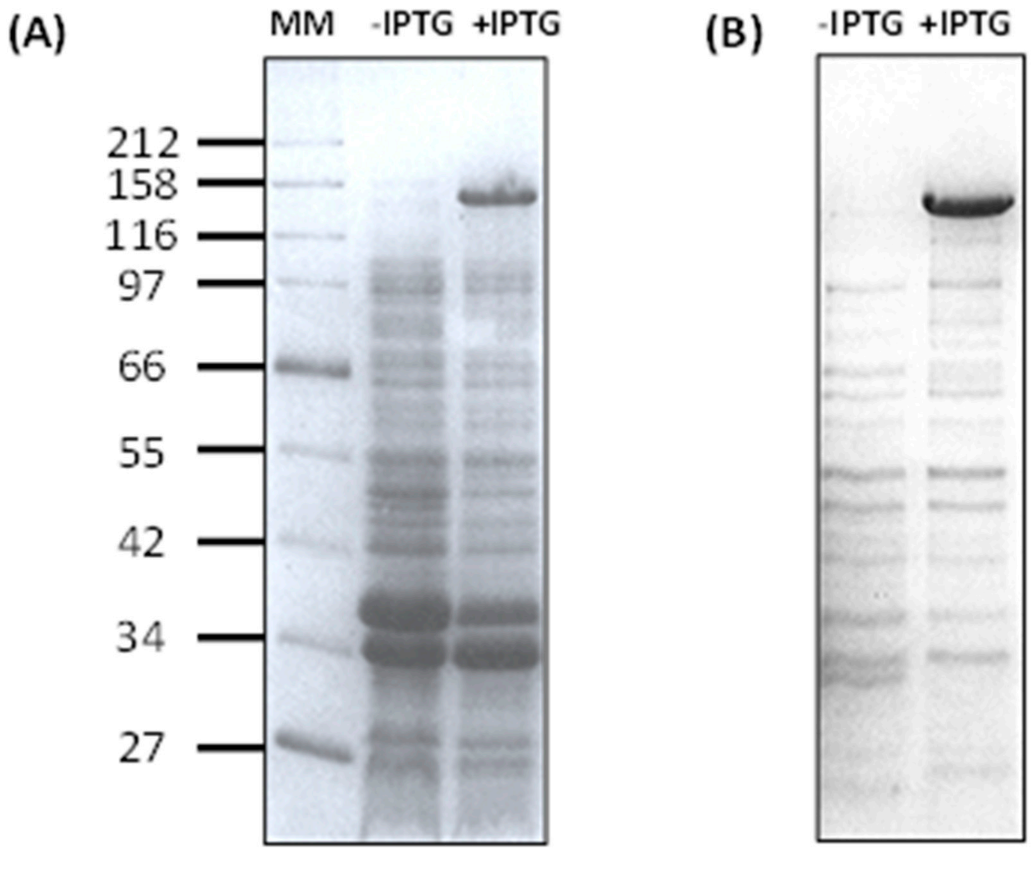

Supplement: Supplementary file 1 [file toxins-17-00084-s001.zip › Supplementary Figure S2.pdf]
